# Impact of Material and Lens Design on Repositioning Surgery of Toric Intraocular Lenses: A Single-Arm Meta-Analysis

**DOI:** 10.1155/2022/6699596

**Published:** 2022-01-27

**Authors:** Jing Wu, Changping Yang, Yan Yin, Linlin Liu, Hui Wang

**Affiliations:** ^1^Department of Ophthalmology, Li Shui Municipal Central Hospital, Lishui, Zhejiang, China; ^2^Gannan Medical University, Ganzhou, Jiangxi, China; ^3^Department of Ophthalmology, Suichang People's Hospital, Lishui, Zhejiang, China; ^4^Second Affiliated Hospital of Shandong First Medical University, Taian, Shandong, China; ^5^Department of Ophthalmology, the First Affiliated Hospital of Gannan Medical University, Ganzhou, Jiangxi, China

## Abstract

**Aim:**

To analyze the pooled incidence rate in repositioning surgery by considering different materials and designs.

**Methods:**

All published studies investigating the repositioning surgery of toric intraocular lenses (IOLs) before September 1, 2020, were searched and evaluated. The R3.5.2 software was used to extract the data, and a single arm meta-analysis was performed.

**Results:**

19 cases from 18 published studies articles were included in the meta-analysis. The pooled incidence rate in repositioning surgery was 2% (*I*^2^ = 53%, *P*_heterogeneity_<0.01). Plate and silicone IOLs had significantly higher incidence rates (6% for each) than loop (2%) and hydrophobic acrylate (2%). Incidence rates of Acrysof, Staar, TECNIS, PhysIOL SA, T-flex 623T, and Microsil 6116TU groups were 1% (95% CI [1%–2%]), 6% (95% CI [4%–9%]), 3% (95% CI [2%–4%]), 1.40% (1/71), 3.03% (1/33), and 4.76% (1/21), respectively.

**Conclusions:**

The pooled incidence rate of repositioning surgery in IOLs was 2%. Materials and designs would be risk factors for the rotational stability of the toric IOLs. Pooled incidence rates of the hydrophobic acrylate and loop group were lower than those of the silicone and plate group. Product identity is the main driver of heterogeneity.

## 1. Introduction

Toric intraocular lenses (IOLs) have been designed to restore visual acuity deteriorated by cataract and correct corneal astigmatism. Clinical studies have reported that toric IOLs have become a safe and effective method to treat cataract patients with preoperative refractive problems [1–3]. However, the precise positioning of the lens in relation to the intended alignment axis is crucial to achieve the intended effect. Toric IOL misalignment by approximately 1° will reduce astigmatic correction by nearly 3.3%, while a 30° misalignment may not correct or increase the astigmatism [4, 5]. Tognetto et al. [6] applied visual information fidelity to analyze the image quality at the IOL rotational step. Previous experiments have illustrated that image quality reduction was observed with a rotation of 30°; subsequently, the images at 45° have the same quality without toric correction.

Thus far, only a small number of studies have examined the rotational stability of different toric IOLs. We aimed to evaluate the postoperative rotation and surgical repositioning of toric IOLs in different materials and designs, through this systematic review and meta-analysis.

## 2. Methods

### 2.1. Search Strategy and Inclusion Criteria

We screened the PubMed, Web of Science, Cochrane Library, ClinicalTrials.gov, CNKI, and Wanfang databases for original articles that were published before September 1, 2020. The searches were conducted using free combinations of the following keywords in both English and Chinese: “toric intraocular lenses,” “toric IOL,” “intraocular lens rotation,” “toric intraocular lens,” “toric phakic intraocular lens,” and “rotation.” Furthermore, we checked the reference lists of the papers selected. Literature search was independently conducted by two researchers (Jing Wu and Changping Yang), followed by resolving of any disagreements via consensus. The included studies met the following inclusion criteria: (1) original research papers regarding the repositioning surgery of toric rotation and (2) randomized controlled clinical trials, nonrandomized clinical trials, cohort studies, uncontrolled cohort studies, and case-control studies. We excluded studies with two or more lens subgroup variations which cannot be combined to obtain their respective incidence rates, along with those that did not satisfy one or more inclusion criteria.

### 2.2. Data Extraction and Study Quality Assessment

Two researchers (Jing Wu and Changping Yang) independently determined whether each study met the inclusion criteria. The following data were subsequently extracted from the included studies using a standardized form: name of the first author, publication year, country, age range, sample size, case, follow-ups, and toric types (shown in Supplementary Table 1). The characteristics of included toric IOLs are shown in Table 1. We used the Newcastle–Ottawa Scale [7] to evaluate the cohort and case-control studies. Quality of the nonrandomized interventional studies was evaluated using the methodological index for nonrandomized studies (MINORS) [8].

### 2.3. Statistical Analyses

Single-rate meta-analyses were carried out using the R statistical software package (version 3.5.2). We combined the experimental data and nonrandomized controlled trials with data from observational studies to perform a single-arm meta-analysis. We used five methods to combine the pooled incidence rate of repositioning surgery of toric intraocular lenses and eventually selected the Freeman–Tukey double arcsine transformations that were closest to normal distribution. Meta-analysis was individually performed for toric intraocular lenses of different materials. All meta-analyses were evaluated for heterogeneity using the chi-square based *I*^2^ test and *Q* test. An interstudy *I*^2^ score <50% or *P* value >0.10 was considered nonheterogeneous; furthermore, we used a fixed-effects model for the meta-analysis. Conversely, we used the random-effects model for meta-analysis in the presence of heterogeneity. The meta-analysis results were based on the forest plot, and the effect size was the combined incidence rates and 95% confidence interval. Subgroup analysis was performed using the *χ*^2^ test, with *P* < 0.05 indicating statistical significance. Additionally, we applied the funnel plot and Egger's linear regression to analyze the publication bias. We also performed the Duval and Tweedie nonparametric “trim and fill” procedure to further assess the possible effects of publication bias in our meta-analysis.

## 3. Results

### 3.1. Characteristics of Included Studies

**Figure 1:**
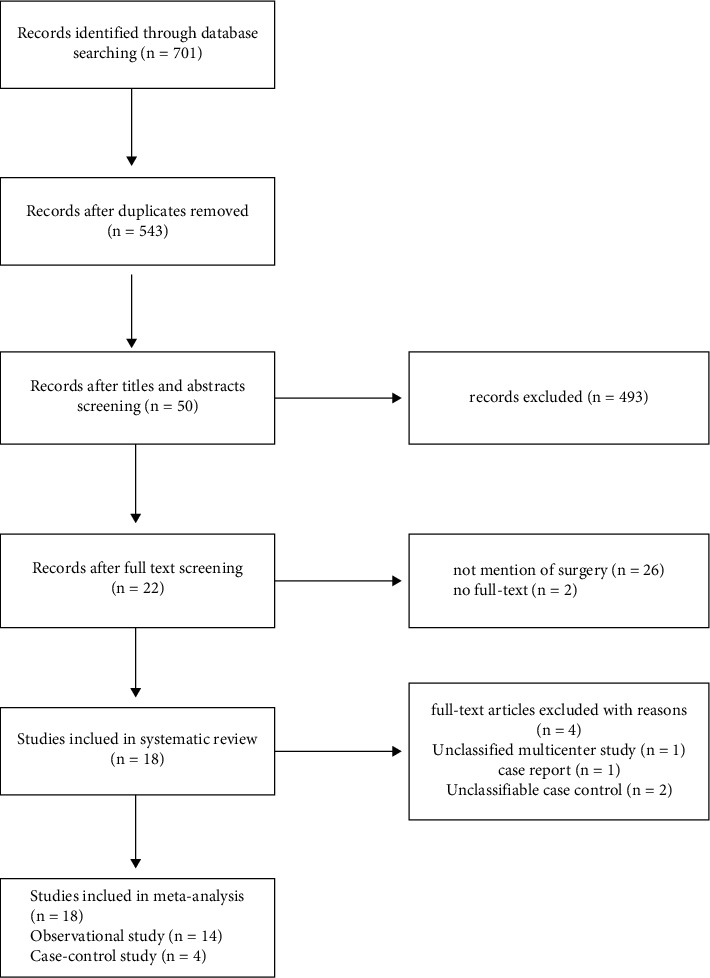
Flowchart demonstrating how the identified published studies were included in the meta-analysis.

After a systematic literature, we identified 701 articles, of which we thoroughly examined 22 full-length articles. We applied the inclusion and exclusion criteria to select 18 studies which included 14 nonrandomized interventional studies [9–21] and 4 cohort and case-control studies [1, 22–24]. The remaining 4 articles were excluded due to the following reason: absence of sufficient information to obtain a definite incidence rate [25–28] (Figure 1).

Characteristics of the included studies have been summarized (shown in the supplementary table see here). We only included the toric IOL subgroup from 3 articles that compared toric and nontoric IOLs [1, 23, 24]. One article with two different datasets was considered as two separate studies [22]. In addition, 4 articles which reported a failure of the relocation surgery were included for subgroup analysis [11, 12, 24, 29]. All included studies were determined to be moderate-to-high-quality studies.

### 3.2. Single-Arm Meta-Analysis

**Figure 2:**
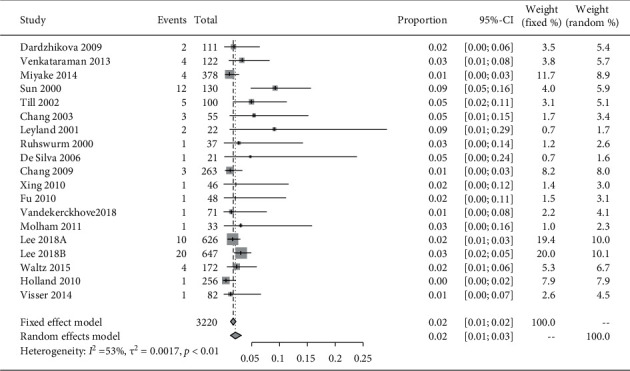
The forest plot displaying the pooled incidence rate of repositioning surgery of toric IOL.

We included 19 cases from the 18 articles in the meta-analysis. The pooled incidence rate of repositioning surgery was 2.0% (95% CI: 1%–3%) (*I*^2^ = 53%, *P*_heterogeneity_<0.01) in toric IOLs. We used the random-effects model for the meta-analysis considering the presence of statistical heterogeneity (Figure 2).

**Figure 3:**
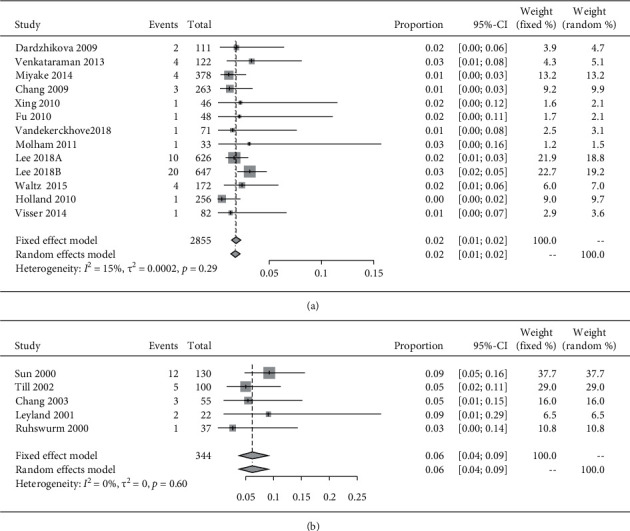
The forest plots displaying the pooled incidence rate of repositioning surgery of (a) loop-haptic toric IOLs and (b) plate-haptic IOLs.

We performed a subgroup analysis of the studies adjusted for haptic designs. The pooled incidence rate of repositioning surgery of plate-haptic toric was significantly higher than that of loop-haptic (2% and 6%, respectively) (OR: 0.264, 95% CI: 0.160–0.436, *P* < 0.001) (Figure 3 and Table 2).

**Figure 4:**
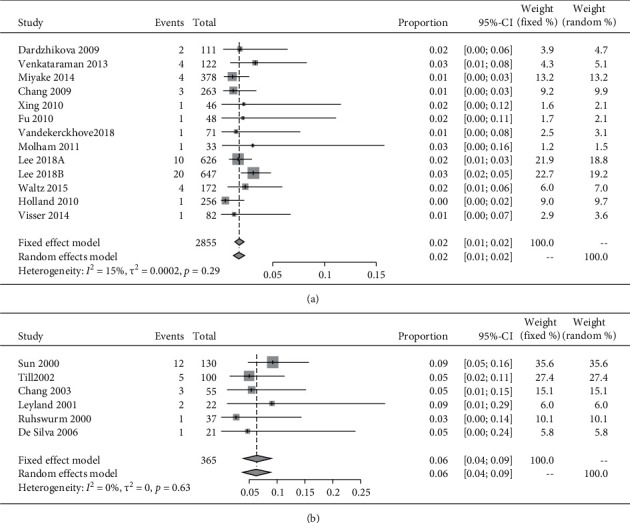
The forest plots displaying the pooled incidence rate of repositioning surgery of (a) hydrophobic acrylic material IOLs and (b) silicone material IOLs.

Furthermore, we performed a subgroup analysis of the studies adjusted based on the materials. Hydrophobic acrylic materials had a lower incidence rate of repositioning surgery of 2% (95% CI: 1–2%), and silicone materials showed a significantly higher incidence rate for the need of a repositioning surgery of 6% (95% CI: 4%–9%) (OR: 0.289, 95% CI: 0.164–0.441, *P* < 0.001) (Figure 4 and Table 2).

**Figure 5:**
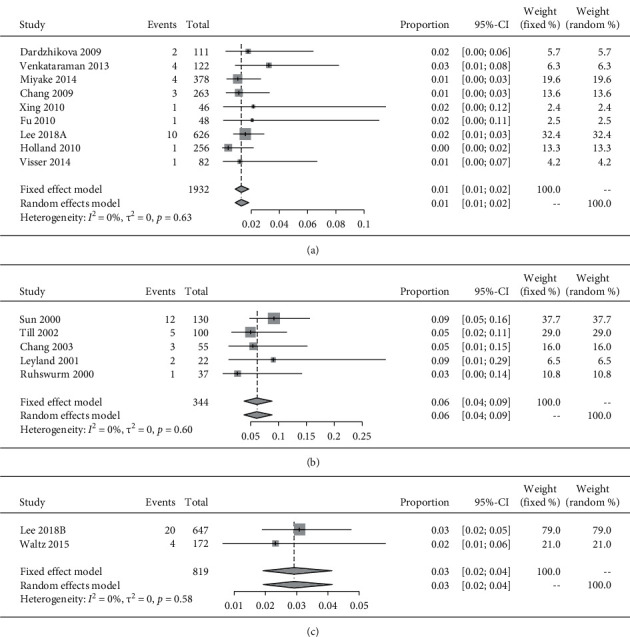
The forest plots displaying the pooled incidence rate of repositioning surgery of (a) Acrysof toric IOLs, (b) Staar IOLs, and (c) TECNIS toric IOLs.

Subgroup analysis was also conducted based on products from different companies. We classified the included toric according to their respective companies or commercial names as Acrysof, Staar, TECNIS, PhysIOL SA, T-flex 623T, and Microsil 6116TU. There were 9 studies in the Acrysof, 5 studies in the Staar, and 2 studies in the TECNIS subgroups. The pooled incidence rate of repositioning surgery was 1% (95% CI: 1%–2%), 6% (95% CI: 4%–9%), and 3% (95% CI: 2%–4%), respectively. Subgroups were compared via the list *χ*^2^ test, which revealed a statistically significant difference (*x*^2^ = 36.383; *P* < 0.001) (Figure 5 and Table 2).

We further used the partitions of the *χ*^2^ method to perform pairwise comparison of multiple sample rates (Table 3). PhysIOL SA, T-flex 623T, and Microsil 6116TU were all included in one study, demonstrating incidence rates of 1.40% (1/71), 3.03% (1/33), and 4.76% (1/21), respectively.

All subgroup comparisons passed the criteria required for the heterogeneity test; subsequently, the fixed-effects models were used for meta-analysis.

### 3.3. Publication Bias

**Figure 6:**
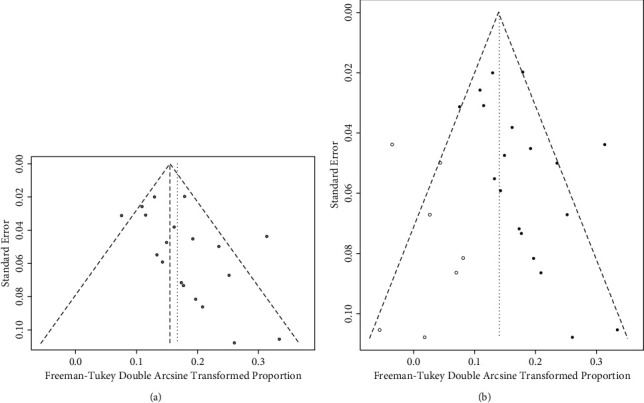
(a) The funnel plot displaying the pooled incidence rate of repositioning surgery of toric IOLs. (b) The filled funnel plot with pseudo-95% CI (the pseudo-95% confidence interval (CI) is computed as part of the analysis that produces the funnel plot).

We used the R software with “metabias,” and the Egger funnel plots are shown in Figure 6. The regression line in the Egger funnel plot did not pass the 0 points, suggesting the presence of publication bias in the literature (Egger's *P* = 0.05184). We performed a sensitivity analysis using the trim and fill method to rectify the same [30], which conservatively imputes the hypothetical negative unpublished studies to mirror the positive studies causing funnel plot asymmetry. After including 7 studies, it produced a symmetrical funnel plot (Figure 6). The pooled incidence rate and 95% CI did not change significantly (1.18%, 95% CI, 0.46%–2.11%). Therefore, the results were considered to be robust and demonstrated a certain degree of reference significance.

## 4. Discussion

Toric IOLs have become an effective tool for patients to eliminate preoperative astigmatism. However, the rotational stability of toric is a significant factor that affects the performance of corrected visual acuity after cataract surgery.

We included of 19 studies comprising 3220 eyes, which showed a 2% pooled incidence rate of repositioning surgery. This incidence observed here was lower than that in previous studies (3–9.2%) [23, 25]. Moreover, Oshika et al. [26] incorporated a large number of case series with 6431 eyes and reported that the overall incidence of repositioning surgery was 0.653%. The lower incidence rate observed in the study may be associated with the distribution of the data. Here, we only included the studies with acrylic foldable toric IOLs; furthermore, all patients with a significant amount of misalignment did not undergo a repositioning surgery. Patients who had no obvious symptoms and those with IOL misalignment and did not consent for further surgical intervention were not included.

Materials used in the studies have demonstrated association with a certain degree rotation of toric IOLs. We observed that performing a subgroup analysis based on the materials demonstrated a significantly higher incidence rate of rotation in silicone IOLs than in hydrophobic acrylate IOLs (OR: 0.289, 95% CI: 0.164–0.441, *P* < 0.001). Lombardo et al. [31] reported that hydrophobic acrylic IOLs showed the highest adhesive properties, followed by hydrophilic acrylic IOLs, PMMA IOLs, and finally silicone IOLs. Linnola et al. [32] also demonstrated that acrylic IOLs had more fibronectin than silicone which had strongest adhesion with capsular bag. Therefore, hydrophobic acrylic IOLs had better rotational stability than the silicone IOLs. In addition, Draschl et al. [33] contrasted two group toric IOLs in the same design with different materials, which subsequently indicated that the hydrophobic acrylic IOLs were better than the hydrophilic IOLs. Here, we also found that the hydrophobic acrylic IOLs demonstrated the best stability.

IOL designs were important to improve the stability of IOL rotation [34]. Evidence showed that the loop-haptic design IOLs had better rotational stability than the plate-haptic (OR: 0.264, 95% CI: 0.160–0.436, *P* < 0.001). Comparing the loop-haptic and plate-haptic IOLs, Patel [35] reported that the plate-haptic tended to rotate more than the loop-haptic design in the early postoperative period. A loop-haptic was prone to a double counterclockwise turn after surgery. Venkataraman et al. [10] also observed that loop-haptic IOLs had excellent stability while early postoperative IOL rotation was more likely to occur only in larger diameter bags.

The Acrysof toric IOLs presented with the best postoperative stability considering the use of different products, followed by TECNIS and Staar IOLs. Acrysof toric IOLs are composed of a hydrophobic acrylate material, which has a particularly strong adhesion. Besides, the loop-haptic demonstrates good memory and softness that can be used to resolve the optical fluctuations caused by shrinkage of the capsular bag. Moreover, it shows a good stability in the capsular bag. Visser et al. [36] reported pooled estimates for the misalignment of more than 10°, indicating the need for a surgical repositioning 3%. Other clinical studies showed that postoperative rotation of Acrysof IOLs is most likely less than 5° [22, 37, 38], with the long AXL, WTR, and oblique astigmatism being risk factors for toric IOLs rotation [39, 40]. TECNIS IOLs have designs and materials similar to those of Acrysof, indicating the presence of a good degree of stability [41]. Hirnschall et al. [42] reported that the average rotation of TECNIS IOLs was 3.27 ± 2.37°. However, we found that the pooled incidence rate of repositioning surgery of TECNIS was higher than that of the Acrysof group (OR: 0.469, 95% CI: 0.269–0.819,  = 0.006). Xue et al. [27] also reported 3 eyes (9%) that required further surgery to rectify the significant IOL rotation. Interestingly, Staar IOLs have a higher postoperative rotation; however, their shorter TF may be considered as one of the risk factors [12, 14, 29]. Chang et al. [13] reported that the TL Staar toric IOLs rotational and repositioning rates were higher than those of TF IOLs. Adequate length is a critical factor to improve the rotational stability of Staar toric IOLs, highlighting the fact that priority should be given to longer IOLs.

Only 4 of 864 eyes demonstrated a failure for repositioning surgery. Among them, Sun et al. [12] reported that the fibrosis of the capsule caused a significant degree of rotation after repositioning, which limited the effect of the position. Xue et al. [27] reported that the reason for the large degree of rotation after surgery was the fact that the patient underwent a preoperative vitrectomy procedure, which decreased the stability of the suspensory ligament. Most clinical studies determined that the IOLs reorientation should be performed within 1 to 3 weeks [26, 29]. Prematurely calibrating the same may rotate the lens again; however, a delay in calibration may become firmly fix the IOLs in the capsule, which upon rotation may cause a zonular rupture [22, 27, 29]. Therefore, good stability can be ensured by selecting appropriate timing of the repositioning procedure and assessing the patient's complications.

Above all, limitations of this study must be considered. First, most studies involved here were observation trials and therefore lacked well-designed randomized double-blind controls. Second, there were no predetermined common criteria for the repositioning surgery. The need for surgical intervention was purely decided by the surgeons responsible for the same, due to which the repositioning surgery was repeated if the patient provided for the same. However, in the absence of the patient's consent, further treatment was not performed. Alternatively, in cases where the patient was dissatisfied with the postoperative corrected vision, regardless of the minimum rotation degree, the case was inadvertently assigned for another survey. Finally, the funnel plot analysis showed some asymmetry that indicated the possibility of sample bias.

## 5. Conclusions

This meta-analysis suggested that the combined incidence of toric IOLs was 2%, which was lower than that reported in the current literature. There is a significant difference in the incidence with the use of different materials, with a lower incidence with regard to the hydrophobic acrylate and the loop-haptic group. Acrysof toric IOLs have better postoperative stability than TECNIS and Staar. Further high-quality studies with more randomized double-blind control designs are needed.

## Figures and Tables

**Table 1:** Characteristics of toric IOLs included in the meta-analysis.

Country	Company	Commercial name	Spherical power	Cylinder power	Design	Haptic	Material
USA	Alcon	Acrysof TIOL SN60TT	+6D∼+34D	+1.5D∼+6.0D	Single-piece	Loop	Hydrophobic acrylic
		Acrysof IQ toric IOL SN6AT	+6D∼+34D	+1.5D∼+6.0D	Aspheric optic	Loop	Hydrophobic acrylic
USA	Abbott Medical Optics	TECNIS	+5D∼+34D	1.00D, 1.50, 2.25, 3.00, 4.00D	Single-piece	Loop	Hydrophobic acrylic
USA	Staar	AA 4203TF/TL	+10D∼+28D	+2D, 3.5D	Single-piece	Plate	Silicone
Germany	Human Optics	Microsil 6116TU	−3D∼+30D	+2D∼+12D	3-Piece	PMMA Z-design	Silicone

**Table 2:** The pooled incidence rate of repositioning surgery with different subgroups.

Variable	Number of articles	Case/total	Pooled estimate [95% CI]	Heterogeneity *I*^2^^*∗*^ (%)	*Q* value	OR (95% CI)	*P* value
Total	19	77/3220	2 [1–3]	53	0.01		

*Haptic*							
Loop	13	53/2855	2 [1–2]	15	0.29	0.264 (0.160–0.436)	≤0.001
Plate	5	23/344	6 [4–9]	0	0.60	1.00	

*Material*							
Silicone	6	24/365	6 [4–9]	0	0.63	1.00	
Hydrophobic acrylic	13	53/2855	2 [1–2]	15	0.29	0.289 (0.164–0.441)	≤0.001

*Products*							
Acrysof	9	27/1932	2 [1–2]	0	0.63	0.198 (0.112–0.349)	0.003
Staar	5	23/344	6 [4–9]	0	0.60	1.00	≤0.001
TECNIS	2	24/819	3 [2–4]	0	0.58	0.421 (0.234–0.757)	0.003
PhysIOL SA	1	1/71					
T-flex 623T	1	1/33					
Microsil 6116TU	1	1/21					

^*∗*^The chi-square test was used for two sample rates and list *χ*^2^ test was used for multiple sample rates. *P* < 0.05 was considered statistically significant.

**Table 3:** Pairwise comparison of multiple sample rates by the partitions of the *χ*2 method.

Subgroup	Sample	No. of samples	*χ* ^2^	*P* value
Acrysof	27	1905	38.011	≤0.001
Staar	23	321		
Total	40	2226		

Acrysof	27	1905	7.428	0.006
TECNIS	24	795		
Total	51	2700		

Staar	23	321	8.811	0.003
TECNIS	24	795		
Total	47	1116		

^*∗*^ *P* < 0.0125 was considered statistically significant.

## Data Availability

All data generated or analyzed during this study are included in relevant published articles.

## Abbreviations

IOLs: Toric intraocular lenses
CI: Confidence interval.

## Supplementary Materials

Supplementary Materials Supplementary Table: characteristics of studies included in the meta-analysis. The data were subsequently extracted from the included studies using a standardized form: name of the first author, publication year, country, age range, sample size, case, follow-ups, and toric types.
