# Supplementary material for: Impact of Material and Lens Design on Repositioning Surgery of Toric Intraocular Lenses: A Single-Arm Meta-Analysis
Source: J Ophthalmol. 2022 Jan 27;2022:6699596. doi: 10.1155/2022/6699596 (PMC8881179; doi:10.1155/2022/6699596)
Supplement: Supplementary Materials — Supplementary Table: characteristics of studies included in the meta-analysis. The data were subsequently extracted from the included studies using a standardized form: name of the first author, publication year, country, age range, sample size, case, follow-ups, and toric types. [file 6699596.f1.docx]

**Supplementary table 1. Characteristics of studies included in the Meta-analysis**

|  | Author publication year | Country | Type of Study | Age range（year） | Sample size（N） | Case（n） | Follow ups(months/weeks  ) | Toric types |
| --- | --- | --- | --- | --- | --- | --- | --- | --- |
| 1 | Dardzhikova 2009 | Canada. | Retrospective observational study. | - | 111 | 2 | 6m | AcrySof TIOL (SN60T) |
| 2 | Venkataraman 2013 | Indian | Prospective observational study | 56±13.88 | 122 | 4 | 12m | AcrySof TIOL (SN60T) |
| 3 | Miyake 2014 | Japan | Prospective observational study | 63.4±16.9 | 378 | 4 | 24m | AcrySof IQ TIOL (SN6AT） |
| 4 | Sun 2000 | Austria | Retrospective observational study | 72 | 130 | 12 | 6.9m | Staar AA4203T TIOL |
| 5 | Till 2002 | USA | Prospective observational study | 74±11 | 100 | 5 | 23±17w | Staar AA4203T TIOL |
| 6 | Chang 2003 | USA | Retrospective observational study | - | 55 | 3 | 1m | Staar AA4203T TIOL |
| 7 | Leyland 2001 | UK | Prospective observational study | 77 | 22 | 2 | 8w | Staar AA4203T TIOL |
| 8 | Ruhswurm 2000 | Austria | Retrospective observational study | 75±9 | 37 | 1 | 20.3±16.6m | Staar AA4203T TIOL |
| 9 | De Silva 2006 | UK | Prospective observational study | 76.1±11.4 | 21 | 1 | 6m | MicroSil 6116TU TIOL |
| 10 | Chang 2009 | USA | Retrospective observational study | - | 263 | 3 | - | AcrySof TIOL (SN60T) |
| 11 | Xing 2010 | China | Retrospective observational study | 65 | 46 | 1 | 3m | AcrySof TIOL (SN60T) |
| 12 | Fu 2010 | China | Retrospective observational study | 58 | 48 | 1 | 6m | AcrySof TIOL |
| 13 | Vandekerckhove 2018 | Switzerland | Prospective observational study | - | 71 | 1 | 12m | PhysIOL SA TIOL (Ankoris/FineVisionPodFT） |
| 14  15  16 | Molham 2011  Lee 2018A  Lee 2018B | UK  USA  USA | Prospective observational study  Retrospective cohort study  Retrospective cohort study | 80.6±8.9  72  72 | 33  626  647 | 1  10  20 | 4m  18m  18m | T-flex 623T TIOL  AcrySof TIOL  TECNIS TIOL |
| 17 | Waltz 2015 | USA and Canada. | Prospective, multicenter, 2-armed, bilateral study | - | 172 | 4 | 6m | TECNIS TIOL |
| 18 | Holland 2010 | USA | Randomized, subject-masked, parallel-group, multicenter study | 71 | 256 | 1 | 12m | AcrySof IQ TIOL (SN6AT） |
| 19 | Visser 2014 | Netherlands | A multicenter, randomized clinical trial | 74 | 82 | 1 | 6m | AcrySof IQ TIOL (SN6AT） |
